# Spotlight on the Compositional Quality of Probiotic Formulations Marketed Worldwide

**DOI:** 10.3389/fmicb.2021.693973

**Published:** 2021-07-20

**Authors:** Diletta Mazzantini, Marco Calvigioni, Francesco Celandroni, Antonella Lupetti, Emilia Ghelardi

**Affiliations:** ^1^Department of Translational Research and New Technologies in Medicine and Surgery, University of Pisa, Pisa, Italy; ^2^Research Center Nutraceuticals and Food for Health-Nutrafood, University of Pisa, Pisa, Italy

**Keywords:** probiotics, compositional quality, viable cells, microbial species, purity, label claims

## Abstract

On the worldwide market, a great number of probiotic formulations are available to consumers as drugs, dietary supplements, and functional foods. For exerting their beneficial effects on host health, these preparations should contain a sufficient amount of the indicated living microbes and be pathogen-free to be safe. Therefore, the contained microbial species and their amount until product expiry are required to be accurately reported on the labels. While commercial formulations licensed as drugs are subjected to rigorous quality controls, less stringent regulations are generally applied to preparations categorized as dietary supplements and functional foods. Many reports indicated that the content of several probiotic formulations does not always correspond to the label claims in terms of microbial identification, number of living organisms, and purity, highlighting the requirement for more stringent quality controls by manufacturers. The main focus of this review is to provide an in-depth overview of the microbiological quality of probiotic formulations commercialized worldwide. Many incongruences in the compositional quality of some probiotic formulations available on the worldwide market were highlighted. Even if manufacturers carry at least some of the responsibility for these inconsistencies, studies that analyze probiotic products should be conducted following recommended and up-to-date methodologies.

## Introduction

Although the term probiotic made its first appearance in 1965, the first universally accepted definition was introduced by the Joint Food and Agriculture Organization of the United Nations (FAO) and the World Health Organization (WHO) in 2001 (FAO/WHO, 2001). For grammatical reasons, this definition was revisited in 2014 and definitively turned into “live microorganisms that, when administered in adequate amounts, confer health benefits on the host” (Hill et al., 2014).

A great number of studies highlight the ability of orally administered probiotics to improve the gut-barrier function, modulate the gut microbiota, enhance host immune response, and exert antimicrobial activities (Bermudez-Brito et al., 2012; Bron et al., 2017; Hu et al., 2017; Maldonado Galdeano et al., 2019). In addition, probiotics can be able to synthetize vitamins, food-degrading enzymes, and molecules contributing to cellular metabolism, thus ameliorating host health (LeBlanc et al., 2017; Oak and Jha, 2019).

Probiotics support physiological bodily functions and may reduce risk or shorten the duration or severity of many diseases. In particular, probiotic drugs are frequently used to prevent or help in the treatment of many gastrointestinal diseases due to infections, microbiome dysbiosis, and gut barrier perturbation. These include traveler, antibiotic-associated, and acute-infectious diarrhea, ulcerative colitis, necrotizing enterocolitis, inflammatory bowel diseases, irritable bowel syndrome, and *Clostridium difficile* and *Helicobacter pylori* infections (Wilkins and Sequoia, 2017). In the last decades, the use of probiotics has been extended to a variety of other disorders, including lactose intolerance, respiratory and urinary infections, asthma, atopic dermatitis, osteoporosis, allergy, metabolic syndromes, as well as liver, neurological, cardiovascular, and autoimmune diseases (Stavropoulou and Bezirtzoglou, 2020).

Microbes used as probiotics must be safe for consumption (FAO/WHO, 2002). To this regard, probiotic microbes intended for human use may have a “generally recognized as safe” (GRAS) notification for specific intended use to the US Food and Drug Administration (FDA, 2019) or the “qualified presumption of safety” (QPS) status (European Food Safety Authority: EFSA, 2007, 2018; Mattia and Merker, 2008). In addition, the antibiotic resistance profile of probiotics should be determined to highlight the presence of acquired resistance genes that can potentially be transmitted to pathogens (FAO/WHO, 2002; Cohen, 2018).

Several lactic acid bacteria (LAB) belonging to the *Lacticaseibacillus, Lactiplantibacillus, Lactobacillus*, *Lentilactobacillus, Levilactobacillus*, *Ligilactobacillus, Limosilactobacillus, Bifidobacterium*, and *Streptococcus* genera are traditionally used as probiotics, constituting the vast majority of commercial products available in the worldwide market (Williams, 2010; Hanchi et al., 2018; Zheng et al., 2020). Spore-forming bacteria of the genus *Bacillus* have progressively taken on, since they can be administered as spores that are extremely resistant to the harsh gastrointestinal conditions (Cutting, 2011; Elshaghabee et al., 2017; Jeżewska-Fra̧ckowiak et al., 2018; Lee et al., 2019). Among yeast, the species *Saccharomyces cerevisiae* var. *boulardii* exhibited a variety of beneficial properties, being adopted as probiotic microbe for several decades (Sen and Mansell, 2020). Several other microorganisms isolated from the human gut (e.g., *Akkermansia muciniphila*, *Faecalibacterium prausnitzii*) have been proposed as next generation probiotic candidates to face up specific diseases (Chang et al., 2019; Zhai et al., 2019).

## Categorization of Probiotics and Notes on Regulation

In different countries, both regulatory aspects that govern probiotics and probiotic categories profoundly differ (for detailed regulatory aspects see Arora and Baldi, 2015; Kumar et al., 2015; de Simone, 2019; Domínguez Díaz et al., 2020; Koirala and Anal, 2021). Probiotics are generally classified as drugs (i.e., medicinal products and pharmaceuticals), dietary supplements (also referred as food supplements), and functional foods depending on the intended use (Halsted, 2003; Sreeja and Prajapati, 2013; de Simone, 2019; Koirala and Anal, 2021). Probiotic drugs are used for the prevention, treatment, and mitigation of human diseases and are subjected to the stringent regulations applied to other drugs, thus requiring approval before marketing and continuous pre- and post-marketing safety and quality controls (Venugopalan et al., 2010; Sreeja and Prajapati, 2013; Kolaček et al., 2017).

Dietary supplements can be administered as tablets, capsules, liquid suspensions, or powders and are intended to complement diet by ensuring the intake of specific dietary components (Taylor, 2004; Domínguez Díaz et al., 2020). In Europe, probiotic food supplements fall under the Food Products Directive and Regulation and each health claim need to be accurately scrutinized by the EFSA before being authorized (de Simone, 2019; Fusco et al., 2021). Good Manufacturing Practice that manufacturers of dietary supplements should follow have been developed (Food Supplements Europe, 2014). In the US, dietary supplements are regulated by the FDA under the Dietary Supplement Health and Education Act (DSHEA) and the Federal Trade Commission (FTC) and need to comply the Good Manufacturing Practice guidelines (Brown, 2017; de Simone, 2019; Fusco et al., 2021).

Although a statutory definition of functional foods does not exist in several countries, these products are intended to provide health benefits being consumed as part of the usual diet (Gul et al., 2016; Domínguez Díaz et al., 2020). This category meets the general laws applicable to foods in many countries (Kumar et al., 2015; Domínguez Díaz et al., 2020; Koirala and Anal, 2021).

## Probiotic Labeling

Probiotic formulations possess specific label requirements that take into account the current laws and regulatory specifications of each country. FAO/WHO reported a list of information that manufacturers are recommended to state on the product label. In particular, labels should clearly indicate the minimum number of each strain until the expiry, the identification of each microorganism contained in the formulation based on the current nomenclature, and the claimed beneficial effects. According to the FAO/WHO guidelines, labels should also contain the suggested serving size to obtain health benefits, the appropriate storage conditions, and corporate contact details (FAO/WHO, 2002).

In 2017, the International Probiotics Association, in partnership with the Council for Responsible Nutrition, added new details to the FAO/WHO indications on the basis of the US regulation and proposed new guidelines for probiotic labeling that worldwide manufacturers were recommended to comply (Council for Responsible Nutrition and International Probiotics Association, 2017). These guidelines were aimed at improving transparency, consistency, and consumer understanding, thus promoting comparisons between different probiotic formulations and improved consumer awareness. Product labels should indicate the quantitative amount of alive microorganisms expressed as colony forming units (CFUs) and the expiration date. For formulations containing multiple species and/or strains, information on the total amount of microorganisms was recommended and, when technically feasible, the amount of each species as well.

## Compositional Quality

Although publications on probiotics rapidly expanded in the last years, most of the studies have been focused on the beneficial properties exhibited by probiotic microbes. A relatively low number of studies analyzed the real-time composition of probiotic formulations that consumers find on the market.

Some reports denouncing poor quality of commercial probiotic products already populated the scientific literature before general guidelines were developed (Gilliland and Speck, 1977; Hamilton-Miller et al., 1996; Yaeshima et al., 1996; Canganella et al., 1997; Hamilton-Miller and Gibson, 1999; Hamilton-Miller et al., 1999; Schillinger, 1999; Hoa et al., 2000). With the introduction of guidelines for probiotics in 2002 (FAO/WHO, 2002), some investigations were conducted to evaluate the compliance between label claims and effective microbial composition, amount of viable cells, and absence of contaminant microorganisms of probiotic formulations available on the market.

In this review, we took into consideration only studies that were published after the introduction of the FAO/WHO guidelines. The probiotic formulations analyzed in different studies were divided into the three category drugs, dietary supplements, and functional foods (Tables 1–3). For simplicity, probiotic formulations indicated as foods with specific medical purposes (FSMP) were included in the category dietary supplements. We decided to incorporate in the tables only products for which name, category, country of origin, and possibly, manufacturer were known. For formulations produced by unmentioned manufacturers, product name and country of origin were used to conduct a careful research for retrieving the missing data. In addition, we did not include commercial products for which the concordance with label claims of amount of living cells, contained species, and purity were not clearly stated by the Authors. For completeness, studies not clearly stating product names, manufacturers, categorization, or origin (further referred to as unidentified probiotics) will be separately discussed in the main text.

**TABLE 1:** Compliance with the label claims of probiotic drugs marketed worldwide: focus on the microbial composition, amount of living cells, and presence of contaminant microorganisms.

Product	Manufacturer	Country	Compliance (composition)	Compliance (amount)	Contaminants	Reference
Benegut	Abbott	India	No	No	Yes	Kesavelu et al., 2020
Bifilac	Tablets India Ltd	India	No	Yes	Yes	Kesavelu et al., 2020
Bifilac GG	Tablets India Ltd	India	Yes	Yes	No	Kesavelu et al., 2020
Biogermin	Union Health S.r.l.	Italy	Yes	Yes	No	Celandroni et al., 2019
Codex	Zambon	Italy	Yes Yes	Yes Yes	No No	De Vecchi et al., 2008 Vecchione et al., 2018
Combiflora	Medopharm	India	No	No	No	Kesavelu et al., 2020
Cyfolac	Karnataka Antib & Pharm Ltd	India	Yes	Yes	No	Kesavelu et al., 2020
Darolac	Aristo Pharmaceuticals Pvt Ltd	India	No	No	No	Kesavelu et al., 2020
Ecogro	Akum Drugs & Pharma	India	No	Yes	Yes	Patrone et al., 2016
Econorm	Dr. Reddy’s Laboratories Ltd	India	Yes	N.D.	No	Kesavelu et al., 2020
Entero Plus	Glaxo India Ltd	India	Yes	Yes	No	Kesavelu et al., 2020
Enterogermina	Sanofi	Italy India	Yes Yes Yes Yes Yes	Yes Yes Yes Yes Yes	No No No No No	De Vecchi et al., 2008 Vecchione et al., 2018 Celandroni et al., 2019 Patrone et al., 2016 Kesavelu et al., 2020
Enterol capsules	Biodiphar	Belgium	Yes	Yes	No	Vanhee et al., 2010
Enterol sachets	Biodiphar	Belgium	Yes	Yes	No	Vanhee et al., 2010
Entromax	Mankind Pharma	India	No	Yes	No	Patrone et al., 2016
GNorm	Nouveau Medicament	India	Yes	N.D.	No	Kesavelu et al., 2020
GutPro	Riata Life Sciences Pvt Ltd	India	Yes	No	No	Kesavelu et al., 2020
Infloran	BERNA	Italy	Yes	No	No	Fasoli et al., 2003
Lacidofil	Merck	Poland	No Yes	Yes Yes	No No	Zawistowska-Rojek et al., 2016 Korona-Glowniak et al., 2019
Lakcid	Biomed	Poland	Yes Yes	Yes Yes	No No	Zawistowska-Rojek et al., 2016 Korona-Glowniak et al., 2019
Ospor	Matrix Pharma	Pakistan	Yes	No	No	Patrone et al., 2016
Pre Pro Kid	Fourrts India Laboratories	India	No	No	No	Kesavelu et al., 2020
Pre Pro Kid L	Fourrts India Laboratories	India	No	No	Yes	Kesavelu et al., 2020
Reflora Z	Sundyota Numandis	India	No	No	No	Kesavelu et al., 2020
Regutol	Alembic Pharmaceuticals Ltd	India	No	Yes	Yes	Kesavelu et al., 2020
Remune Al	Sundyota Numandis	India	No	No	No	Kesavelu et al., 2020
SPORLAC	Sanzyme Ltd	India	Yes^∗^	No	No	Kesavelu et al., 2020
Super Flora GG	Sundyota Numandis	India	Yes	No	No	Kesavelu et al., 2020
Tufpro	Virchow Biotech Pvt. Ltd.	India	No	No	Yes	Patrone et al., 2016
ViBact	USV	India	No	Yes	Yes	Kesavelu et al., 2020
Vizylac	Torrent Pharmaceuticals Ltd	India	Yes^∗^	No	No	Kesavelu et al., 2020

*N.D., not definable.*

*^∗^Incorrect nomenclature.*

**TABLE 2:** Compliance with the label claims of probiotic dietary supplements marketed worldwide: focus on the microbial composition, amount of living cells, and presence of contaminant microorganisms.

Product	Manufacturer	Country	Compliance (composition)	Compliance (amount)	Contaminants	Reference
40 + Acidophilus	Solgar Laboratories	The Netherlands	No	N.D.	No	Temmerman et al., 2003a
4lacti	Norfarm	Poland	No	No	No	Korona-Glowniak et al., 2019
Acidolac^#^	Polpharma	Poland	Yes	Yes	No	Zawistowska-Rojek et al., 2016
Acidophi Kidz	Nature’s Plus	United States	No	No	No	Drago et al., 2010
Acidophilus Pearls	Enzymatic	United States	Yes	Yes	No	Drago et al., 2010
Acidophilus Plus	Quest Vitamins	United Kingdom	No	N.D.	No	Temmerman et al., 2003a
Acidophilus Probiotic	Nature’s bounty	United States	No	No	Yes	Drago et al., 2010
Aciforce	Biohorma	Netherlands	No No	N.D. N.D.	No No	Temmerman et al., 2003a Temmerman et al., 2003b
Align Digestive Care	Procter & Gamble	United States	Yes	No	No	Drago et al., 2010
Antedia	Will-Pharma	Belgium	Yes	Yes	No	Vanhee et al., 2010
Asecurin	Aflofarm	Poland	No No	No No	No No	Zawistowska-Rojek et al., 2016 Korona-Glowniak et al., 2019
Bacilac	THT	Belgium	No	N.D.	No	Temmerman et al., 2003b
Bactisubtil	Synthelabo Belgium	Belgium	Yes Yes	N.D. N.D.	No No	Temmerman et al., 2003a Temmerman et al., 2003b
Beneflora	ORTIS	Belgium	No	N.D.	No	Temmerman et al., 2003a
Bifidus complex	Biover	Belgium	No	N.D.	Yes	Temmerman et al., 2003a
Bififlor	Eko-Bio	Netherlands	No	N.D.	No	Temmerman et al., 2003b
Bifilact	Fidia Farmaceutici	Italy	No	No	No	Fasoli et al., 2003
BioGaia^#^	Ewopharma	Poland	Yes	Yes	No	Zawistowska-Rojek et al., 2016
BioGaia Protectis	Ewopharma	Poland	Yes	Yes	No	Korona-Glowniak et al., 2019
Biolactine family	Sella	Italy	No	No	Yes	Celandroni et al., 2019
BioPro Reuteri straws	Unknown	South Africa	Yes	Yes	No	Elliot and Teversham, 2004
BioPro Reuteri tablets	Unknown	South Africa	Yes	Yes	No	Elliot and Teversham, 2004
Biopron 9	Valosun	Poland	No	No	No	Korona-Glowniak et al., 2019
Biotyk	Lekam	Poland	No Yes	No No	No No	Zawistowska-Rojek et al., 2016 Korona-Glowniak et al., 2019
Boulardi-Sanifort	Sanifort Pharma	Belgium	Yes	Yes	No	Vanhee et al., 2010
Coloflor^#^	Oleofarm	Poland	Yes	No	No	Zawistowska-Rojek et al., 2016
Colon C	A-Z Medica	Poland	No	Yes	No	Zawistowska-Rojek et al., 2016
Combiforte capsules	Bioflora CC	South Africa	No	Yes	No	Elliot and Teversham, 2004
Culturelle	CAG Functional Foods	United Kingdom	Yes	N.D.	No	Temmerman et al., 2003a
Culturelle	Amerifit	United States	Yes	Yes	No	Drago et al., 2010
Culturelle sachets	Unknown	South Africa	No	Yes	Yes	Elliot and Teversham, 2004
Culturelle tablets	Unknown	South Africa	No	No	No	Elliot and Teversham, 2004
Daily Fiber and Prob.	Walgreens	United States	No	No	Yes	Drago et al., 2010
Diaclo	Sandoz	Germany	Yes	Yes	No	Vanhee et al., 2010
Dicoflor	Dicopharm	Italy	Yes Yes	No Yes	No No	De Vecchi et al., 2008 Vecchione et al., 2018
Dicoflor^#^	Vitis Pharma	Poland	Yes	Yes	No	Korona-Glowniak et al., 2019
Dicoflor 30 Kid^#^	Vitis Pharma	Poland	Yes	Batch dep.	No	Zawistowska-Rojek et al., 2016
Dicoflor 60^#^	Vitis Pharma	Poland	Yes	Yes	No	Zawistowska-Rojek et al., 2016
Enterelle	Bromatech	Italy	Yes	Yes	No	Vecchione et al., 2018
Enteriphar	Teva	Israel	Yes	No	No	Vanhee et al., 2010
Enterofermenti family	SB Pharma C	Italy	Yes	No	No	Celandroni et al., 2019
Enterolactis	SOFAR	Italy	Yes	No	No	De Vecchi et al., 2008
Enterolactis Plus	SOFAR	Italy	Yes	Yes	No	Vecchione et al., 2018
Enterolife	Paladin Pharma	Italy	Yes	No	No	Celandroni et al., 2019
Ercéflora Supra	Sanofi Aventis	France	Yes	N.D.	No	Vanhee et al., 2010
Ferzym Plus	Specchiasol	Italy	No	No	Yes	Celandroni et al., 2019
Floraviva	Tredi Farmaceutici	Italy	No	No	No	Fasoli et al., 2003
GiSol	Metagenics	Belgium	Yes	Yes	No	Vanhee et al., 2010
Ido-Form Kid	Ferrosan	Poland	Yes	Yes	No	Zawistowska-Rojek et al., 2016
Iladian	Aflofarm	Poland	No	No	No	Zawistowska-Rojek et al., 2016
Imutis	Trenker Pharmaceutical	Belgium	Yes	Yes	No	Vanhee et al., 2010
Infantiforte capsules	Unknown	South Africa	Yes	Yes	No	Elliot and Teversham, 2004
LaciBios Femina	Asa	Poland	Yes	Yes	No	Zawistowska-Rojek et al., 2016
Lacidar	Tantus	Poland	No	No	No	Zawistowska-Rojek et al., 2016
Lacidozone	Ozone Laboratories	Poland	No	No	No	Zawistowska-Rojek et al., 2016
Lacium Zdrovit	Zdrovit	Poland	Yes	No	No	Zawistowska-Rojek et al., 2016
Lactèol	Menarini Benelux	Belgium	No	N.D.	No	Temmerman et al., 2003a
Lacteol Forte capsules	Unknown	South Africa	No	No	No	Elliot and Teversham, 2004
Lacteol Forte sachets	Unknown	South Africa	No	No	No	Elliot and Teversham, 2004
Lactimum	Biorès	Belgium	No	N.D.	Yes	Temmerman et al., 2003a
Lactiv up	Farma-Projekt	Poland	Yes	No	No	Zawistowska-Rojek et al., 2016
Lactò Piu‘	Recordati OTC	Italy	Yes	No	No	Celandroni et al., 2019
Lacto5	Biosan	Italy	No	No	No	Fasoli et al., 2003
Lactoflorene Plus	Montefarmaco OTC	Italy	Yes	No	No	Vecchione et al., 2018
Lactoral	Biomed	Poland	No	No	No	Zawistowska-Rojek et al., 2016
Lactovita capsules	Unknown	South Africa	No	No	No	Elliot and Teversham, 2004
Latopic^#^	IBSS Biomed	Poland	No	Yes	No	Zawistowska-Rojek et al., 2016
Life Top Straw	BioGaia Biologics	Sweden	Yes	N.D.	No	Temmerman et al., 2003a
Linex Forte	Lek Pharmaceuticals	Poland	Yes	Yes	No	Zawistowska-Rojek et al., 2016
Multi-billion dophilus	Solgar Laboratories	Netherlands	No	N.D.	No	Temmerman et al., 2003a
MultiTab ImmunoKid	Ferrosan	Poland	Yes	Yes	No	Zawistowska-Rojek et al., 2016
Natrol Prob. Intest.	Natrol	United States	No	Yes	Yes	Drago et al., 2010
Neolactoflorene	Montefarmaco OTC	Italy	No	Yes	No	Fasoli et al., 2003
Novaflora	Pharmaphood	Belgium	No	N.D.	No	Temmerman et al., 2003a
Nucleogermina	Pharmaelle	Italy	No	Yes	Yes	Celandroni et al., 2019
Nutriplant	Agropharm	Poland	Yes	Batch dep.	No	Zawistowska-Rojek et al., 2016
Omniflora Akut	Novartis	Switzerland	Yes	Yes	No	Vanhee et al., 2010
Optibac-*S. boulardii*	Wren Laboratories	United Kingdom	Yes	Yes	No	Vanhee et al., 2010
Oslonka normal	Apotex	Poland	Yes	Yes	No	Korona-Glowniak et al., 2019
Phillip’s Colon Health	Bayer	United States	Yes	Yes	No	Drago et al., 2010
Prévite acidophilus	Unknown	Unknown	No	N.D.	Yes	Temmerman et al., 2003a
Probiosan	Nutrisan	Belgium	Yes*	N.D.	No	Temmerman et al., 2003a
Probiotic Immunity	New Chapter Organics	United States	No	Yes	Yes	Drago et al., 2010
Proflora	Chefaro	Belgium	No No	N.D. N.D.	No No	Temmerman et al., 2003a Temmerman et al., 2003b
Progermila	Chemist’s Research	Italy	No	Yes	Yes	Celandroni et al., 2019
Progermila bambini	Chemist’s Research	Italy	No	Yes	Yes	Celandroni et al., 2019
Prolife	Zeta Farmaceutici	Italy	Yes	Yes	No	Vecchione et al., 2018
Provag^#^	Biomed	Poland	No	Yes	No	Zawistowska-Rojek et al., 2016
Psyllium actif	Biover	Belgium	No	N.D.	Yes	Temmerman et al., 2003a
Reuflor	Italchimici	Italy	Yes	Yes	No	Vecchione et al., 2018
Reuterin/Reuflor	Noos/Italchimici	Italy	Yes	Yes	No	De Vecchi et al., 2008
*S. boulardii*	Supersmart	Portugal	Yes	Yes	No	Vanhee et al., 2010
Sacchiflora	3D Pharma	Belgium	Yes	N.D.	No	Vanhee et al., 2010
Sanprobi IBS	Sanum Poland	Poland	Yes	No	No	Zawistowska-Rojek et al., 2016
Super Acidophilus	GNC	United States	No	No	Yes	Drago et al., 2010
Super Probiotic Comp.	GNC	United States	No	No	Yes	Drago et al., 2010
Superior Probiotics	BioGaia Biologics	Sweden	Yes	N.D.	No	Temmerman et al., 2003a
Sustenex	Ganaden Biotech	United States	Yes	Yes	No	Drago et al., 2010
Triflora	Farmapia	Poland	Yes	Batch dep.	No	Zawistowska-Rojek et al., 2016
Trilac^#^	Krotex	Poland	Yes	No	No	Korona-Glowniak et al., 2019
Ultra Prob. compl. 25	GNC	United States	No	Yes	Yes	Drago et al., 2010
Ultralevure	Biocodex	France	Yes	N.D.	No	Vanhee et al., 2010
VSL#3	Ferring Farmaceutici	Italy	Yes Yes	Yes Yes	No No	Vecchione et al., 2018 Mora et al., 2019
Wapiti Darmbalans	Emonta b.v.	Netherlands	Yes	Yes	No	Vanhee et al., 2010
Yovis	AlfaSigma	Italy	No Yes	No Yes	No No	Fasoli et al., 2003 Vecchione et al., 2018
ZirFos	Alfa Wasserman	Italy	No	No	No	De Vecchi et al., 2008

*N.D., not definable.*

*^∗^Incorrect nomenclature.*

*^#^Food for specific medical purposes.*

**TABLE 3:** Compliance with the label claims of probiotic functional foods marketed worldwide: focus on the microbial composition, amount of living cells, and presence of contaminant microorganisms.

Product	Manufacturer	Country	Compliance (composition)	Compliance (amount)	Contaminants	Reference
ABC	Sitia YOMO	Italy	Yes	N.D.	No	Fasoli et al., 2003
Actimel	Danone	France	Yes* Yes	N.D. N.D.	No No	Temmerman et al., 2003a Temmerman et al., 2003b
Actimel orange	Danone	France	Yes*	N.D.	No	Temmerman et al., 2003a
Active-più	Parmalat	Italy	Yes	N.D.	No	Fasoli et al., 2003
Activia	Danone	Italy France Russia	Yes* Yes N.D.	N.D. N.D. Yes	No No No	Fasoli et al., 2003 Temmerman et al., 2003b Astashkina et al., 2014
Almighurt	Almighurt	Germany	Yes	N.D.	No	Temmerman et al., 2003a
B’A fruits	B’A	France	No	N.D.	No	Temmerman et al., 2003a
B’A vanille	B’A	France	No	N.D.	No	Temmerman et al., 2003a
Benecol	McNeil Cons Nutritionals	United Kingdom	No	N.D.	No	Temmerman et al., 2003a
BI’AC	TMA	Germany	No	N.D.	No	Temmerman et al., 2003a
BIO abricot	Danone	France	No	N.D.	No	Temmerman et al., 2003a
BIO framboise	Danone	France	No	N.D.	No	Temmerman et al., 2003a
Bio Snac’	Danone	France	No	N.D.	No	Temmerman et al., 2003a
Biogarde Hafvol Naturel	Strothmann	Germany	No	N.D.	No	Temmerman et al., 2003a
Biogarde plus (naturel)	Almhof	Netherlands	No	N.D.	No	Temmerman et al., 2003a
Biomild drink	Mona	Netherlands	No	N.D.	No	Temmerman et al., 2003a
Biospega	Spega	Italy	Yes	N.D.	No	Fasoli et al., 2003
Dannon	Dannon	Russia	N.D.	Yes	No	Astashkina et al., 2014
Fitness Quark	Onken	Germany	No	N.D.	No	Temmerman et al., 2003a
Fysiq	Mona	Netherlands	No	N.D.	No	Temmerman et al., 2003a
Gefilus	Valio	Finland	Yes	N.D.	No	Temmerman et al., 2003a
Imunele	Wimm Bill- Dan	Russia	N.D.	Yes	No	Astashkina et al., 2014
Joghurt Mild Gartenfrutch	Bremerland	Germany	No	N.D.	No	Temmerman et al., 2003a
Kinderyoghurt mild	J. Bauer KG	Germany	No	N.D.	No	Temmerman et al., 2003a
Kyr	Giglio	Italy	Yes	N.D.	No	Fasoli et al., 2003
Lactus Nature	Carrefour	France	Yes*	N.D.	No	Temmerman et al., 2003a
Lc1	Nestlè	Germany	Yes	N.D.	No	Temmerman et al., 2003a
Mio	Nestlè	Italy	Yes	N.D.	No	Fasoli et al., 2003
Natumild	Natuur Hoeve	Netherlands	No	N.D.	No	Temmerman et al., 2003a
Pomogayka	Nestle	Russia	N.D.	Yes	No	Astashkina et al., 2014
Procult Drink	Alois Müller	Germany	No	N.D.	No	Temmerman et al., 2003a
Provie	Skåne Mejerier	Sweden	Yes	N.D.	No	Temmerman et al., 2003b
Teddy	Fattoria Scaldasole	Italy	Yes	N.D.	No	Fasoli et al., 2003
Vifit Drink	Mona	Netherlands	No	N.D.	No	Temmerman et al., 2003a
Vitamel	Campina	Netherlands	No	N.D.	No	Temmerman et al., 2003b
Weight Watchers Bifidus	Senoble	France	No	N.D.	No	Temmerman et al., 2003a
Yakult	Yakult	Netherlands	Yes* Yes	N.D. N.D.	No No	Temmerman et al., 2003a Temmerman et al., 2003b

*N.D., not definable.*

*^∗^Incorrect nomenclature.*

### Microbial Composition

The identity of microbes (i.e., at genus, species, and strain level) included in commercial probiotic formulations should be stated by the manufacturer on the product label and follow the current accepted nomenclature (FAO/WHO, 2002; Council for Responsible Nutrition and International Probiotics Association, 2017). To this regard, Weese analyzed the labels of 21 dietary supplements intended for human use to verify label’s accuracy (Weese, 2003). The author showed that nine products reported adequate label information in terms of microbial composition with only two indicating microbes at the strain level. On the other products, microorganisms were indicated with a name not concordant with the actual nomenclature or misspelled (Weese, 2003). Similarly, label analysis of the top 10 probiotic formulations sold on the Indian market revealed that seven did not specify the contained species, but only gave an unsuitable explanation (i.e., Lactic Acid *Bacillus*; Ghattargi et al., 2018).

In this study, we also evidenced that the labels of some formulations, particularly functional foods, did not comply general guidelines for probiotic labeling, since not indicating the contained microbes or only reporting a general and inaccurate description (e.g., “LAB,” “probiotics,” “lactic ferment,” “lactic acid bacillus,” “live probiotic AB cultures,” “active bifidus,” “special live cultures,” “viable LAB,” “bifidus essensis,” “life yeast”) (Fasoli et al., 2003; Temmerman et al., 2003a,b; Elliot and Teversham, 2004; Masco et al., 2005; Theunissen et al., 2005; Lin et al., 2006; Perea Vélez et al., 2007; Angelakis et al., 2011; Chen et al., 2014; Kesavelu et al., 2020). In addition, we found that some preparations only indicated the contained microbes at the genus level, thus resulting in disagreement with the FAO/WHO guidelines (Fasoli et al., 2003; Temmerman et al., 2003a,b; Masco et al., 2005; Theunissen et al., 2005; Lin et al., 2006; Perea Vélez et al., 2007; Chen et al., 2014; Yonkova Marinova et al., 2019).

Regarding probiotic drugs, the microbial composition of 31 products available in Europe (Italy, Belgium, and Poland), India, and Pakistan is reported in Table 1 (column 4). Only four products among these 31 (Codex, Enterogermina, Lacidofil, Lakcid) were analyzed by independent studies, with three of them showing concordant results. In fact, *Lactobacillus acidophilus* declared to be contained in Lacidofil was detected only in one study by species-specific PCR (Korona-Glowniak et al., 2019) but not in the other, which used biochemical methods and matrix assisted laser desorption/ionization-time of flight mass spectrometry (MALDI-TOF MS) (Zawistowska-Rojek et al., 2016). Overall, our analysis highlighted that 17 formulations (56.67%) contained the species stated on the labels. *Bacillus coagulans* in two products (SPORLAC, Vizylac) was labeled with an obsolete nomenclature (i.e., *Lactobacillus sporogenes*). Thirteen formulations (43.33%) did not comply the label claims. In particular, six formulations (Darolac, Ecogro, Entromax, Pre Pro Kid L, Regutol, Tufpro) contained more species than those declared, while three (Combiflora, Reflora Z, Remune Al) lacked one or more species. In addition, Benegut, Bifilac, Pre Pro Kid, and Vibact possessed different species than those labeled (Table 1, column 4).

Regarding dietary supplements, the microbial composition of a total of 106 products marketed around the world is reported in Table 2 (column 4). Byotik and Yovis were analyzed in two studies by using different methods, but the recovered species were found to be concordant with the label claims only in one study each. The discordant results obtained for Yovis could depend on the different years in which the studies were performed (Fasoli et al., 2003; Vecchione et al., 2018). In fact, it can not be excluded the product underwent changes in 15 years and the manufacturer’s controls had increased over time. Aciforce, Asecurin, Bactisubtil, Dicoflor (Dicopharm), Proflora, and VSL#3 were also analyzed by independent studies with concordant results. Globally, among 104 products, 56 (53.85%) contained the same species declared on the labels, with the exception of *Lactobacillus crispatus* in Probiosan that was indicated with an obsolete nomenclature. The other 48 products (46.15%) were not compliant for the microbial composition, since lacking one or more species or containing more or different species than those declared on the labels (Table 2, column 4).

As regards products categorized as functional foods, a total of 37 formulations is reported in Table 3 (column 4). For three products (Dannon, Imunele, Pomogayka), the correspondence between contained species and label information could not be established, since the identification of microbes isolated from these products was only performed at the genus level (Astashkina et al., 2014). Actimel, Activia, and Yakult were analyzed by independent studies, all showing concordant results with label information. Globally, the microbial composition was concordant with the label claims for 15 functional foods (44.12%), with 5 of them reporting an obsolete nomenclature (Actimel, Actimel Orange, Activia, Lactus Nature, Yakult). In the other 19 products (55.88%), no concordance between microbial composition and labeled information was found.

As regards studies on unidentified probiotics, Weese reported concordant results with label claims in only 2 of 5 products intended for human use (Weese, 2002). Analysis of the microbial composition of 58 products obtained from 13 Countries indicated that many formulations were mislabeled (Masco et al., 2005). Similarly, a study conducted on 10 products sold in Europe indicated that three were not concordant with the label claims and four were concordant at the species level but contained different strains than those labeled (Coeuret et al., 2004). Aureli et al. (2010) verified the compliance to the label claims of 41 dietary supplements sold in Italy showing that 20 were constituted by the species reported on the labels. Toscano et al. (2013) investigated the microbial composition of 24 dietary supplements available on the Italian market by using PCR, pyrosequencing of the 16S rRNA gene, and biochemical methods. They showed that 21 products were compliant with the label claims. Nevertheless, seven of these supplements did not contain all the labeled microbes in the amount declared by manufacturers (Toscano et al., 2013). Di Pierro et al. (2019) evaluated the compositional quality of nine formulations declaring to contain alive *L. crispatus* alone or in combination with other species. The presence of *L. crispatus* in the formulations was investigated by using selective media and by species-specific PCR (Di Pierro et al., 2019). The authors found that all products contained *L. crispatus* by culture-independent methods, but only six formulations carried living cells (Di Pierro et al., 2019). The identification of species declared to be contained in five Italian oral products revealed concordant microbial composition to the label claims for four formulations (Blandino et al., 2016). Identification of species contained in 13 French functional foods by MALDI-TOF MS, PCR and sequencing of *tuf* and 16S rRNA genes revealed different species than those stated for two products (Angelakis et al., 2011). The analysis of different lots of a probiotic formulation intended for preterm infant in The Netherlands evidenced the absence of one or more species claimed on the label depending on the tested lot (Vermeulen et al., 2020). The microbial composition of five Polish medicinal products was analyzed by biochemical methods (Szajewska et al., 2004). This study showed that only three products completely complied the labeled information in terms of composition, while the other two contained other species than those declared (Szajewska et al., 2004). Investigation of 26 Bulgarian dietary supplements by two-step multiple PCR indicated that none contained all the species claimed on the labels (Yonkova Marinova et al., 2019).

By using PCR-based denaturing gradient gel electrophoresis (DGGE) and species-specific PCR to decipher the microbial composition of 20 products commercialized in South Africa, Theunissen et al. (2005) found that 54.6% of functional foods and 33.3% of dietary supplements contained the microorganisms stated on the labels.

In a study aimed at evaluating the composition of seven functional foods available on the Columbian market, the authors showed that the content of many dairy products did not comply the label claims (Perea Vélez et al., 2007). Compliance for microbial composition was found for 1 of 5 products available in the US (Drisko et al., 2005). The analysis of the microbial compositions of 14 dietary supplements commercialized in the US by terminal restriction fragment length polymorphism (T-RFLP) and species-specific PCR revealed that 12 products contained additional species than those declared (Marcobal et al., 2008). By studying the bacterial diversity of 16 *Bifidobacterium*-containing products available in the US market, Lewis et al. (2016) showed that only 1 product completely fulfill the label claims. The other formulations lacked one or more *Bifidobacterium* species and/or contained additional species. In addition, a pill-to-pill and lot-to-lot variation was highlighted by the authors for most formulations (Lewis et al., 2016). In a complex study aimed at evaluating the microbiological quality of 52 dietary supplements sold in North Carolina, the authors showed that 30 products effectively contained the labeled species (Morovic et al., 2016). By culture-independent metagenomic sequencing of 10 dietary supplements marked in the US, Patro et al. (2016) showed that five products were mislabeled (Patro et al., 2016).

The analysis of 28 products available on the Chinese market revealed that some preparations respected the labeled species (Chen et al., 2014). Lastly, 4 of 17 products marketed in China were not compliant with the labeled information for microbial composition (Ullah et al., 2019).

### Amount of Viable Cells

According to the guidelines for probiotics, manufacturers are required to declare the amount of microbes contained in commercial formulation by the expiration date. In the analysis of labels of 21 products for human use conducted by Weese, 16 were shown to report the contained amount of cells (Weese, 2003). In this analysis of the published data, the amount of cells was not labeled for two drugs (Econorm, GNorm; Table 1, column 5) and three dietary supplements (Ercèflora Supra, Sacchiflora, Ultralevure; Table 2, column 5). Two dietary supplements (Acidophilus Probiotic Gold, Natrol Probiotic Intestinal) declared the total amount of microbes at the production time and not at the expiration date (Table 2, column 5).

As regards studies on unidentified probiotics, the microbial amount was not declared for one Italian dietary supplement, 4 European functional foods, 5 Indian products, 4 products sold in Taiwan, and 15 dairy products available on the Chinese market (Coeuret et al., 2004; Lin et al., 2006; Chen et al., 2014; Ghattargi et al., 2018; Di Pierro et al., 2019).

The majority of multispecies or multistrain products were found specifies on the labels only the total amount of microorganisms. In fact, species/strain amount declaration was only found for six drugs (Bifilac, Pre Pro Kid, Pre Pro Kid L, Reflora Z, Regutol, Vibact; Table 1) and five dietary supplements (Biolactine family, Infloran, Ferzym Plus, Floraviva, Yovis; Table 2). Three drugs and two dietary supplements sold in China, one functional food available in South Africa, as well as two drugs and nine supplements marketed in Italy indicated the specific amount of the contained species or strain (Theunissen et al., 2005; Aureli et al., 2010; Toscano et al., 2013; Chen et al., 2014; Di Pierro et al., 2019; Ullah et al., 2019).

Microbial counts in probiotic formulations were conventionally performed by the plate count method. This culture-based analysis may underestimate the real number of alive microbes if methodologies which are not up to current standards were used. Recommended methods for the analysis of probiotics in foods and supplements have been published (Champagne et al., 2011; Schoeni, 2015). In the analysis of probiotic formulations the use of an appropriate medium, period, and temperature for rehydration, homogenization procedure, plating medium, and incubation time, temperature, and redox level should be adopted (Champagne et al., 2011; Schoeni, 2015). Regarding the evaluation of the amount of viable cells compared with the number declared on the label, we found that 15 out of 29 probiotic drugs (51.72%) were compliant (Table 1, column 5). Among them, all the products investigated in different studies (Codex, Enterogermina, Lacidofil, Lakcid) provided similar results. The other 14 probiotic drugs (48.28%) contained less microbes than those stated on the labels.

As regards to dietary supplements (Table 2, column 5), microbial viability was not definable for 22 products, since the amount of cells was not declared. In addition, contrasting outputs were obtained for Dicoflor and Yovis in different studies (Fasoli et al., 2003; Vecchione et al., 2018) and some variations due to different product batches were observed for Dicoflor 30 Kid, Nutriplant, and Triflora (Table 2, column 5). Among the remaining 79 supplements, 44 (55.70%) were compliant for the number of cells. The total CFU amount obtained for 35 products (44.30%) was lower than the number declared on the labels. In particular, the analysis of Asecurin and Byotik in two independent studies led to the same incongruent results (Zawistowska-Rojek et al., 2016; Korona-Glowniak et al., 2019).

As shown in Table 3 (column 5), the compliance with the labeled amount of microbes could not be established for 33 out of 37 functional foods, since the amount of cells was not declared (Fasoli et al., 2003; Temmerman et al., 2003a,b). The remaining four products analyzed for microbial viability (Activia, Dannon, Imunele, Pomogayka) contained the amount of cells declared by manufacturers on labels.

As regards studies on unidentified probiotics, Masco et al. (2005) showed that many of the investigated formulations did not contain viable cells. An incongruent microbial amount was found in 5 among 10 investigated European products (Coeuret et al., 2004). Thirty among 46 analyzed Italian products contained the amount of living microbes declared on the labels (Aureli et al., 2010; Blandino et al., 2016). Among 24 Italian products tested by Toscano et al. (2013), 10 were found to contain an incorrect amount of cells. On a total of nine Italian analyzed formulations, only two contained the amount of viable cells declared by manufacturers (Di Pierro et al., 2019). All the Polish probiotic formulations included in the study by Szajewska et al. (2004) complied with the label claims for content of viable cells. The analysis of the content of living microbes in 26 supplements sold in Bulgaria revealed that for 10 there was concordance with the label claims (Yonkova Marinova et al., 2019).

By analyzing the amount of viable cells of five dietary supplements available in South Africa, three of them resulted to contain a lower microbial load than that labeled (Brink et al., 2005). Determination of viable *Bifidobacterium* conducted in 58 yogurts from 7 manufacturers sold in North Carolina revealed that 14 did not contain viable cells (Ibrahim and Carr, 2006). Morovic et al. (2016) found that 17 of 52 supplements had a CFU count drastically below the labeled amount. On a total of 10 formulations analyzed, one was found to contain a lower amount of viable cells than that declared by the manufacturer (Patro et al., 2016).

In one study aimed at investigating the presence of LAB in eight probiotic products available in Taiwan, the authors found that two products did not contain the labeled amount of LAB since no viable cells were recovered from these formulations (Lin et al., 2006). For 28 Chinese products, concordance with the labeled number of total living microbes was evidenced for 13 products (Chen et al., 2014). Nevertheless, the authors showed that other 12 products in which the contained amount of cells was not labeled met the minimum quantitative requirement of living cells established in China (Chen et al., 2014). In addition, Ullah and coauthors showed that 5 among 17 tested Chinese dietary supplements contained a number of viable cells that was not concordant with the amount declared on the labels. In particular, four products were found to contain a lower microbial load and no viable cells were detected in one product (Ullah et al., 2019). Among four analyzed dietary supplements available in Bangladesh, none contained the labeled amount of cells (Begum et al., 2015).

### Microbiological Purity

Commercial probiotic formulations should be free of pathogenic organisms (further reported as contaminants) that can constitute a health risk for humans. While all the analyzed functional foods were shown to lack contaminants (Table 3, column 6), 7 drugs (22.58%; Table 1, column 6) and 17 dietary supplements (16.04%; Table 2, column 6) contained potential pathogens.

As regards to drugs, the presence of *Bacillus thuringiensis* was revealed in Benegut and Regutol, while *Enterococcus hirae* was detected in Bifilac, Pre Pro Kid L, and Vibact (Table 1). Interestingly, while DGGE detected *Bacillus cereus, Alcaligenes faecalis*, *Xanthomonas* spp., and *Lysinibacillus* spp. in Tufpro, culture-dependent analysis identified only *B. cereus* in the product, thus indicating the presence of viable cells of this organism (Table 1). Culture-independent DGGE analysis of Ecogro (Table 1) revealed several contaminants, e.g., *B. cereus*, *Bacillus licheniformis*, *Acinetobacter* spp., *Xanthomonas*/*Pseudomonas* spp. *Staphylococcus*/*Lysinibacillus* spp. Regarding dietary supplements, high loads of *B. cereus* (about 1.0 × 10^10^ CFU/dose) and *Lysinibacillus fusiformis* (about 1.3 × 10^11^ CFU/dose) were detected in Progermila bambini (Table 2). In addition, *B. cereus* was found to contaminate 1 unidentified dietary supplement (4.5 × 10^6^ CFU/g) available on the Italian market (Aureli et al., 2010). The products Biolactine family and Ferzym Plus were found to contain more than 1.0 × 10^3^ CFU/dose of *B. licheniformis* and *Bacillus badius*, respectively (Table 2). Super Probiotic Complex was contaminated by *Bacillus lentus* (9.0 × 10^9^ CFU/dose) and *Bacillus* spp. (1.0 × 10^2^ CFU/dose). Contamination by *Bacillus* spp. was also found in Daily Fiber and Probiotics (3.5 × 10^2^ CFU/dose) and Natrol Probiotic Intestinal (1.3 × 10^8^ CFU/dose). Drago et al. (2010) showed the presence of 1.0 × 10^2^ CFU/dose of *Staphylococcus* spp. in Acidophilus Probiotic Gold and a mold in Ultra Probiotic Complex. *L*. *fusiformis* was detected in high amount (about 7.0 × 10^11^ CFU/dose) in Progermila and *Acinetobacter baumannii* (about 1.2 × 10^11^ CFU/dose) was isolated from Nucleogermina (Table 2).

*Enterococcus faecium* was the most common contaminant found in dietary supplements. The microorganism was found in Bifidus complex, Culturelle sachets, Lactimum, Prévite acidophilus, and Psyllium actif (Table 2). *E. faecium* was also detected in high amount in Probiotic Immunity (4.2 × 10^9^ CFU/dose), Super Acidophilus (4.1 × 10^5^ CFU/dose), and Super Probiotic Complex (9.9 × 10^4^ CFU/dose) (Table 2). Additionally, the microbe was found in several unidentified products. In particular, *E. faecium* was revealed in one US and two Italian dietary supplements, as well as in most of the Chinese products analyzed by Chen et al. (2014; Toscano et al., 2013; Patro et al., 2016). The presence of *E. faecium* and *Enterococcus faecalis* was denounced in some batches of a Dutch product for infants (Vermeulen et al., 2020).

Other potential pathogens such as *B. cereus*, *B. licheniformis*, *Enterobacter cloacae*, *Enterobacter* spp., *Staphylococcus epidermidis*, *Klebsiella* spp. and *Serratia* spp. were revealed by DGGE analysis in some Chinese probiotics (Chen et al., 2014). Nevertheless, none of these microbes were isolated from the products by using culture-dependent methods, thus suggesting that they could be present in a killed form (Chen et al., 2014). Microorganisms belonging to the genus *Weissella* were isolated from some products marketed in Bulgaria (Yonkova Marinova et al., 2019). Lastly, contamination of a dietary supplement for preterm newborns by *Rhizopus oryzae* was evidenced by culture-dependent methods (Vallabhaneni et al., 2015).

## Discussion

According to their definition, probiotics must be alive and administered in adequate amount to benefit host health (FAO/WHO, 2001, 2002; Hill et al., 2014). The administration of probiotic preparations containing lower amounts of living microbes may lead to reduced or even absent beneficial effect (Ouwehand, 2017). The daily amount of living probiotics should derive from *in vitro* and *in vivo* experiments and be opportunely determined for each strain and product. Therefore, a universal dose to administer is not established *a priori* for all products. However, some countries (e.g., Italy) require that the daily dose of probiotic formulations contains at least 10^9^ CFU (Ministero della Salute, 2018). Manufacturers are recommended to clearly state the total content of microbes on the product label and this amount should be guaranteed until the expiration date at the declared handling and storage conditions (FAO/WHO, 2002; Guarner et al., 2012; Council for Responsible Nutrition and International Probiotics Association, 2017; World Gastroenterology Organisation [WGO], 2017).

The analysis of commercial probiotic products included in this review highlighted a critical scenario. Many probiotics, particularly functional foods, do not indicate the contained amount of microbes on the label and discrepancies between label information and real content emerged. Even if manufacturers carry at least some of the responsibility for these incongruences, it should be mentioned that the included studies have adopted different methodologies that were sometimes not rigorous and not adherent to recommended methods for the analysis of probiotic products. In fact, papers published before 2015 could have suffered of the lack of guidelines for good practices (Schoeni, 2015) and the adopted methods led to erroneous (underestimated) or incomplete results. It should be mentioned that many studies analyzed products off the shelf, and did not store them until the expiration (“best before”) date prior to analysis. It could be that some products judged to be “compliant” were not at the expiration date, particularly if the manufacturers allow storage at room temperature. Thus many conclusions in Table 2 are only “presumptively compliant.”

Among the formulations that declare the total CFU number, more than 40% included in the category drugs and dietary supplements carried a lower amount of viable cells than stated. In the analyzed studies, determination of the amount of living microbes was performed by the plate count method and the number of cells for dose or gram of product was generally provided (Jackson et al., 2019). Although some culture-independent technologies (e.g., flow cytometry, direct imaging and enumeration, nucleic acid amplification techniques) for quantifying the total microbial content (i.e., alive and dead cells) of probiotic formulations are available, the plate count method currently represents the gold-standard applied in probiotic industry for quality controls (International Standards Organisation [ISO], 2006; International Standards Organisation [ISO], 2010; Davis, 2014; Jackson et al., 2019). However, some limits on the use of the plate count method for the analysis of probiotic formulations have been evidenced. In addition to the fact that not all microbes are culturable by traditional methods and that different microorganisms can require extremely variegate cultural conditions, this technique is unable to detect viable but non-culturable (VBNC) cells that can be generated by the stressful manufacturing procedures (Davis, 2014; Jackson et al., 2019). To obtain the correct enumeration of microbes contained in probiotic products, both plate-count and culture-independent techniques should be applied, as performed in a limited number studies included in this review (Vanhee et al., 2010; Morovic et al., 2016; Patro et al., 2016; Ullah et al., 2019). In culture-dependent analyses, enumeration of different species in multi-species formulations can be critical. To overcome this limit, selective media are commonly used to discriminate species with different metabolic features (Fasoli et al., 2003; Elliot and Teversham, 2004; Aureli et al., 2010; Drago et al., 2010; Toscano et al., 2013; Chen et al., 2014; Di Pierro et al., 2019; Korona-Glowniak et al., 2019; Yonkova Marinova et al., 2019; Kesavelu et al., 2020). Nevertheless, this aim remains challenging, particularly for *Lactobacillus* and *Bifidobacterium* species. In fact, some selective media can impair growth of the target microbe or allow growth of other microorganisms, thus resulting not completely selective (Roy, 2001; Davis, 2014; Hayek et al., 2019; Margolles and Ruiz, 2021).

The viability of probiotic microbes in the finished product is influenced by manufacturing, packaging, and handling. To this regard, overage amounts of microbes are commonly included by manufacturers in probiotic supplements to guarantee the presence of the labeled dose until the expiration date (Fenster et al., 2019). In addition, probiotic products are often produced in a dried or microencapsulated form to ensure stability of microbes over time (Fenster et al., 2019; Grumet et al., 2020). Adequate packing is also crucial to prevent humidification, which can affect cell viability (Fenster et al., 2019).

An additional factor that affects the stability of microbes is the duration of the product shelf life. In fact, while for functional foods the expiration date is generally measured in days or months, for dietary supplements and drugs it is commonly considerably longer (i.e., up to 24 months) (Fenster et al., 2019). Different studies demonstrated a progressive and time-related decrease in the number of viable cells in probiotics investigated at different times before the expiry (De Vecchi et al., 2008; Toscano et al., 2013; Blandino et al., 2016). Stability controls should be performed by manufacturers to guarantee the presence of living cells, since some probiotic organisms can enter in a viable but non-culturable status or lose viability (Forssten et al., 2011; Jackson et al., 2019).

Beneficial health effects of probiotics are generally species- and often strain-specific (Hill et al., 2014; Vitetta et al., 2017). Therefore, the correct identification of microorganisms contained in commercial probiotic formulations is of crucial importance (FAO/WHO, 2002; Council for Responsible Nutrition and International Probiotics Association, 2017).

In this work, we highlighted that more than 40% of the formulations included in the category drugs, dietary supplements, and functional foods do not comply the label claims for microbial composition. Analyzing all the investigated papers over time and looking at the percentages of compliant formulations, no substantial improvement in the quality of probiotic products was found (Supplementary Table 1). Nevertheless, due to the fact that many of the analyzed papers were published many years ago, we cannot exclude the current situation of marketed probiotics is improved.

The evidenced lack of concordance between label claims and microbial composition could be due to manufacturer-dependent biases and flaws in quality controls. The FAO/WHO guidelines recommended to identify microbes included in probiotic formulations by using molecular techniques, such as DNA-DNA hybridization or 16S rRNA gene sequencing (FAO/WHO, 2002). In a comprehensive study analyzing 213 microbial cultures intended for production of probiotic formulations, more than 28% were incorrectly identified for the application of unsuitable identification methods (Huys et al., 2006).

It is remarkable to underline that the use of different methods for the identification of microbes can lead to divergent results, particularly for multispecies products. Contrasting results in the composition of the same probiotic brands were evidenced in studies using different methods (Fasoli et al., 2003; Zawistowska-Rojek et al., 2016; Vecchione et al., 2018; Korona-Glowniak et al., 2019). In addition, we cannot exclude that some of the analyzed papers could have produced erroneous conclusions, since not applying rigorous and up-to-date methodologies in the analysis of probiotic formulations. In most of the included studies, the microbial composition of probiotic products was analyzed by a combination of culture-dependent methods (e.g., biochemical tests, MALDI-TOF MS, species-specific PCR, sodium dodecyl sulfate polyacrylamide gel electrophoresis, sequencing of target genes, microsatellite-based typing) applied to microbes isolated from the formulations (Temmerman et al., 2003a; Coeuret et al., 2004; Szajewska et al., 2004; Perea Vélez et al., 2007; De Vecchi et al., 2008; Aureli et al., 2010; Drago et al., 2010; Vanhee et al., 2010; Blandino et al., 2016; Zawistowska-Rojek et al., 2016; Vecchione et al., 2018; Celandroni et al., 2019; Korona-Glowniak et al., 2019; Vermeulen et al., 2020; Yonkova Marinova et al., 2019). However, culture-dependent methods are strictly bound to the ability of the investigators to grow *in vitro* all microbes contained in probiotic products and to phenotypically discriminate colonies belonging to different species. This aim is particularly challenging for multispecies formulations and can result in the inability to detect some species, as humbly reported in some studies (Temmerman et al., 2003a; Drago et al., 2010; Vecchione et al., 2018). In addition, culture-dependent methods fail in the detection of VBNC cells that do not grow on selective media and some biochemical tests (e.g., API system) have been shown to misidentify closely related species and identify *Bifidobacterium* only at the genus level (Blandino et al., 2016; Zawistowska-Rojek et al., 2016). Similarly, sequencing of the 16S rRNA gene can result unable to distinguish closely related species that share an almost identical sequence of this gene (Fusco et al., 2021). On the other hand, culture-independent techniques (DGGE, T-RFLP, species-specific PCR, high-throughput sequencing, shotgun metagenomic sequencing) directly applied to the whole probiotic products were primarly adopted for microbial identification in some papers (Elliot and Teversham, 2004; Drisko et al., 2005; Theunissen et al., 2005; Marcobal et al., 2008; Chen et al., 2014; Morovic et al., 2016; Mora et al., 2019). These methods lead to detect all the species effectively contained in the products (including VBNC) but are unable to establish if the identified species are viable. Although some probiotic cells have been shown to maintain their beneficial properties also in a dead form (Taverniti and Guglielmetti, 2011; de Almada et al., 2016), probiotics are required to be alive to benefit host health (FAO/WHO, 2001). Considering the limits of culture-dependent and culture-independent techniques, we believe that a combination of both methods should be reasonably applied to definitely decipher the microbial composition of commercial probiotic products, as performed in some of the included studies (Fasoli et al., 2003; Temmerman et al., 2003b; Masco et al., 2005; Angelakis et al., 2011; Toscano et al., 2013; Lewis et al., 2016; Patro et al., 2016; Patrone et al., 2016; Di Pierro et al., 2019; Ullah et al., 2019; Kesavelu et al., 2020). Recently, whole genome sequence analysis has been proposed as a promising method for the identification of microbes contained in probiotic formulations (Fusco et al., 2021).

Purity of commercial probiotic formulations represents a major issue in product testing and quality controls. The introduction of potential pathogenic microorganisms in probiotics can accidentally occur during the entire manufacturing and handling steps and constitute a dangerous health threat, particularly for susceptible individuals. A case of fatal gastrointestinal mucormycosis in a preterm infant due to the assumption of probiotic dietary supplement contaminated by the mold *Rhizopus oryzae* has been reported (Vallabhaneni et al., 2015). Although some *Enterococcus* strains are conventionally used as probiotics, FAO/WHO recommended to keep careful attention in the inclusion of *Enterococcus* microbes in commercial formulations. In fact, some strains can exhibit transmissible vancomycin resistance, acquire other resistance genes, and show a certain degree of pathogenic potential (FAO/WHO, 2001, 2002; Ben Braïek and Smaoui, 2019).

*Enterococcus hirae*, *E. faecium*, and *E. faecalis* were found to contaminate some probiotic products, particularly dietary supplements. However, Temmerman et al. (2003a) declared than none of *E. faecium* strains isolated in their study as contaminant was resistant to vancomycin. Due to their marked antibiotic resistance and ability to cause urinary tract and wound infections, endocarditis, and bacteremia, *E. faecium* and *E. faecalis* are well known as important nosocomial pathogens (Madsen et al., 2017; Gao et al., 2018; Ben Braïek and Smaoui, 2019; Zhou et al., 2020). Differently, *E. hirae* is generally considered a zoonotic pathogen and is rarely isolated from clinical samples derived from humans. Nevertheless, some studies highlighted this microorganism as responsible with urinary tract infections, endocarditis, and bacteremia (Bourafa et al., 2015; Dicpinigaitis et al., 2015; Ebeling and Romito, 2019; Pinkes et al., 2019; Winther et al., 2020).

Other products resulted to be contaminated with high *Bacillus* loads. Although some *Bacillus* strains are traditionally used as probiotics, others are sufficiently equipped of virulence determinants and cause infections in humans (Cutting, 2011; Celandroni et al., 2016; Elshaghabee et al., 2017; Jeżewska-Fra̧ckowiak et al., 2018; Lee et al., 2019). In fact, *B. cereus* is well known as causative agent of two-types of food poisoning diseases. While the emetic syndrome is due to the ingestion of food contaminated by the pre-formed cereulide, the diarrhoic syndrome follows the consumption of food containing high doses of *B. cereus* spores. Once reached the gut, spores germinate and vegetative cells produce some enterotoxins that are responsible of the symptomatology (Ehling-Schulz et al., 2019). In addition, many other opportunistic infections such as endophthalmitis, endocarditis, bacteremia, as well as wound, respiratory, urinary, and central nervous system infections have been associated with this organism (Bottone, 2010; Celandroni et al., 2016; Ehling-Schulz et al., 2019). The ability of *B. thuringiensis* to cause food-poisoning diseases, periodontitis, wound infections, and bacteremia in humans has occasionally been reported (Celandroni et al., 2014). *B. licheniformis* can be responsible of serious diseases such as peritonitis, food poisoning, and bacteremia, particularly in immunocompromised patients (Park et al., 2006; Celandroni et al., 2016). *B. badius* and *B. lentus* are generally not considered human pathogens, but some strains of *B. lentus* have been shown to possess many *B. cereus* virulence factors (Beattie and Williams, 1999).

*Lysinibacillus fusiformis* was also detected in some products. This species can behave as opportunistic pathogen in humans, particularly immunocompromised patients, being responsible of a variety of infections (Wenzler et al., 2015). Isolation of the opportunistic nosocomial pathogen *A. baumannii*, potentially causing severe infections, was also reported (Antunes et al., 2014; Harding et al., 2018; Celandroni et al., 2019).

In conclusion, in our review of the published data we found a great number of inconsistencies in the compositional quality of many probiotic formulations available on the worldwide market. In general, no improvement in the quality of products over time was evidenced (Supplementary Table 1). Several discrepancies were also observed for probiotic drugs, although this category is subjected to different quality controls compared to dietary supplements and functional foods (de Simone, 2019). As regards quality of probiotic formulations, the ESPGHAN Working Group for Probiotics and Prebiotics already published a society paper stressing the need to improve quality controls for commercial formulations (Kolaček et al., 2017). More recently, another international expert panel emphasized the requirement of more transparency by manufacturers on the quality of probiotics (Jackson et al., 2019). We also believe manufacturers should be imposed to perform more accurate quality controls and adopt innovative methods for producing preparations that are microbiologically pure and qualitatively and quantitatively compliant with the label claims. Scientists who examine compliance of products should wait until the expiration date to carry out the analysis and at the storage temperature indicated on the label; if there is no mention of the need to refrigerate the product during storage, it should be tested after storage at room temperature. Furthermore, it should be stressed that the use of adequate methods for enumerating and identifying microbes contained in commercial formulations by the investigators is crucial to obtain an accurate and objective overview of the product quality. In addition, it should be underlined the urgent need for more specific and shared regulatory guidelines that govern the global market of these widely used products.

## Author Contributions

DM and EG reviewed the literature and wrote the manuscript. MC, FC, and AL contributed to the analysis and discussion. All the authors revised the manuscript and approved its submission.

## Conflict of Interest

The authors declare that the research was conducted in the absence of any commercial or financial relationships that could be construed as a potential conflict of interest.
