# Supplementary material for: Spotlight on the Compositional Quality of Probiotic Formulations Marketed Worldwide
Source: Front Microbiol. 2021 Jul 20;12:693973. doi: 10.3389/fmicb.2021.693973 (PMC8329331; doi:10.3389/fmicb.2021.693973)
Supplement: Supplementary file 1 [file Table_1.DOCX]

| **Year** | **Number of products compliant with the label claims** | **Number of products non-compliant with the label claims** | **Percentage of non-compliance** | |
| --- | --- | --- | --- | --- |
| 2020 | 6 | 14 | | 70.0% |
| 2019 | 8 | 13 | | 61.9% |
| 2018 | 9 | 1 | | 10.0% |
| 2016 | 12 | 18 | | 60.0% |
| 2014 | 4 | 0 | | 0% |
| 2010 | 18 | 10 | | 35.7% |
| 2008 | 3 | 3 | | 50.0% |
| 2004 | 3 | 6 | | 66.7% |
| 2003 | 24 | 41 | | 63.1% |

**Supplementary Table S1: Number of products compliant and non-compliant with the label claims during the years.**
